# Network meta-analysis of first-line R-CHOP-based regimens in MYC/BCL2 double-expressor diffuse large B-cell lymphoma

**DOI:** 10.3389/fimmu.2026.1832980

**Published:** 2026-06-23

**Authors:** Hao Cheng, Lin Zeng, Xuanzhang Li, Qing Ke, Jie Sun, Baoping Guo, Xiaohong Tan, Hong Cen, Chengcheng Liao

**Affiliations:** 1 Department of Hematology/Oncology, Guangxi Medical University Cancer Hospital, Nanning, Guangxi, China; 2 Department of Thyroid and Breast Surgery, The First Affiliated Hospital of Guangxi University of Chinese Medicine, Nanning, Guangxi, China; 3 State Key Laboratory of Targeting Oncology, Guangxi Medical University, Nanning, Guangxi, China

**Keywords:** diffuse large B-cell lymphoma, double-expressor lymphoma, MYC/BCL2, network meta-analysis, polatuzumab vedotin, R-CHOP, tucidinostat

## Abstract

Double-expressor lymphoma (DEL), characterized by concurrent overexpression of MYC and BCL2 without gene rearrangements, represents 20%–30% of DLBCL and confers inferior outcomes with standard R-CHOP. However, no head-to-head trials comparing novel R-CHOP-based regimens specifically in DEL exist. We conducted a frequentist network meta-analysis to compare four modified R-CHOP regimens in treatment-naïve DEL patients. PubMed, EMBASE, Cochrane Library, and Web of Science were searched through December 2025. The efficacy analysis included four studies (CAVALLI, DEB, POLARIX, Randomised Evaluation of Molecular guided therapy for Diffuse Large B-cell lymphoma with Bortezomib (REMoDL-B); 1,016 DEL patients) evaluating Pola-R-CHP, Ven-R-CHOP, VR-CHOP, and CR-CHOP versus R-CHOP in a star-shaped network in which all interexperimental comparisons are purely indirect through the common R-CHOP comparator. For progression-free survival (PFS), Pola-R-CHP was the only regimen achieving statistical significance (HR = 0.65, 95% CI = 0.45–0.94; SUCRA = 76.1%), reducing progression risk by 35%. A supplementary Bayesian analysis confirmed this finding (posterior probability of HR < 1: 90.2%). No regimen significantly improved overall survival (OS). CR-CHOP showed the most favorable OS trend (HR = 0.77, 95% CI = 0.53–1.13; SUCRA = 69.8%). Safety analysis from intention-to-treat populations revealed that Ven-R-CHOP significantly increased grade 3–4 thrombocytopenia (OR = 17.24), neutropenia (OR = 3.42), and anemia (OR = 3.29), whereas Pola-R-CHP demonstrated a safety profile comparable to R-CHOP (any grade 3–4 AE: OR = 1.04). Sensitivity analyses excluding CAVALLI (historical control design), using alternative DEL definitions for REMoDL-B, and excluding REMoDL-B confirmed robustness. Pola-R-CHP may offer a favorable benefit–risk profile in DEL; however, this is based on indirect, subgroup-level evidence of moderate-to-low certainty, and prospective DEL-specific trials are needed for confirmation. The study protocol was registered on INPLASY (registration number: INPLASY202630036; DOI: 10.37766/inplasy2026.3.0036).

## Introduction

Diffuse large B-cell lymphoma (DLBCL) is the most common aggressive non-Hodgkin lymphoma subtype, accounting for approximately 25% of all cases. Since the early 2000s, rituximab plus cyclophosphamide, doxorubicin, vincristine, and prednisone (R-CHOP) has remained the standard first-line treatment, achieving long-term remission in approximately 60% of patients (1–3). However, molecular heterogeneity within DLBCL results in substantial variability in treatment response (4, 5).

The 2016 World Health Organization classification introduced the concept of double-expressor lymphoma (DEL), defined by concurrent immunohistochemical (IHC) overexpression of MYC (≥ 40%) and BCL2 (≥ 50%) proteins without corresponding gene rearrangements (i.e., MYC and/or BCL2 translocations) (6). DEL accounts for 20%–30% of newly diagnosed DLBCL cases and is associated with significantly inferior outcomes compared with non-DEL DLBCL when treated with R-CHOP (7–9). The synergistic oncogenic cooperation between MYC and BCL2 promotes tumor cell proliferation while simultaneously inhibiting apoptosis, creating a particularly treatment-resistant phenotype (10).

Several phase 2 and 3 trials have evaluated novel agents combined with R-CHOP in DLBCL, with DEL subgroup analyses reported. The POLARIX trial demonstrated that polatuzumab vedotin plus R-CHP (Pola-R-CHP) significantly improved progression-free survival (PFS) in the overall population, with a particularly pronounced benefit in the DEL subgroup (11, 12). The CAVALLI study evaluated venetoclax plus R-CHOP (Ven-R-CHOP) in a single-arm phase 2 design with historical controls from the GOYA trial (13). The DEB trial assessed tucidinostat (chidamide) plus R-CHOP (CR-CHOP) in a randomized phase 3 trial specifically enrolling DEL patients (14). The REMoDL-B trial evaluated bortezomib plus R-CHOP (VR-CHOP) in a randomized phase 3 trial (15). The PHOENIX trial tested ibrutinib plus R-CHOP but enrolled exclusively nongerminal center B-cell (non-GCB) patients, limiting its comparability for efficacy analysis (16).

A critical clinical challenge is the absence of treatment guidelines specifically designed for DEL patients and the lack of head-to-head trials directly comparing these novel R-CHOP-based regimens. Most existing evidence derives from subgroup analyses of broader DLBCL trials, and substantial heterogeneity in trial design, patient selection criteria, efficacy endpoints, and follow-up duration precludes reliable cross-study comparison through conventional pairwise meta-analysis (17, 18).

Network meta-analysis (NMA) enables simultaneous comparison of multiple treatments through a common comparator, even in the absence of direct head-to-head evidence (17). We conducted a systematic review and frequentist NMA to compare the relative efficacy and safety of novel R-CHOP-based regimens in treatment-naïve DEL patients, to inform evidence-based treatment selection for this high-risk population.

## Methods

### Study design and registration

This systematic review and NMA were conducted following the Preferred Reporting Items for Systematic Reviews and Meta-Analyses (PRISMA) extension statement for NMA (PRISMA-NMA) (19). This systematic review and network meta-analysis were registered on the International Platform of Registered Systematic Review and Meta-analysis Protocols (INPLASY), with registration number INPLASY202630036 (DOI: 10.37766/inplasy2026.3.0036), and conducted in accordance with the PRISMA-NMA guidelines.

The completed PRISMA-NMA checklist is provided in **Supplementary Table 1**.

### Literature search

Two independent investigators systematically searched PubMed, EMBASE, Cochrane Library, and Web of Science from database inception through December 2025. The search strategy, reviewed by a medical librarian, combined MeSH terms and free-text keywords including “diffuse large B-cell lymphoma”, “double-expressor”, “MYC”, “BCL2”, “R-CHOP”, “polatuzumab vedotin”, “venetoclax”, and “bortezomib”. Conference abstracts from the American Society of Hematology and European Hematology Association annual meetings (2020–2024) were hand-searched.

### Eligibility criteria

Inclusion criteria were as follows: (1) randomized controlled trials (RCTs) or prospective interventional studies with concurrent or historical controls; (2) treatment-naïve DLBCL patients with DEL subgroup data available; (3) interventions comprising R-CHOP or modified R-CHOP regimens; (4) reporting of hazard ratios (HRs) with 95% confidence intervals (CIs) for PFS and/or overall survival (OS), or Kaplan–Meier curves from which these could be extracted; and (5) minimum follow-up of 12 months. Exclusion criteria were as follows: (1) observational studies without controls, case reports, or case series; (2) relapsed/refractory DLBCL; (3) inability to extract DEL subgroup survival data; (4) duplicate publications; and (5) non-English and non-Chinese publications. Lenalidomide-based regimens (R2CHOP) were excluded because the ROBUST trial showed no PFS benefit in non-GCB DLBCL and did not report IHC-defined DEL subgroup survival data, and E1412 was a phase 2 signal-seeking study with a DEL subgroup too small to report separate HRs.

### Interventions and outcomes

Five studies were identified: CAVALLI, DEB, POLARIX, PHOENIX, and REMoDL-B. PHOENIX enrolled exclusively non-GCB patients, which is inconsistent with the other four trials; therefore, it was excluded from the efficacy NMA but included in the safety analysis. The efficacy of NMA was thus compared to four regimens (Pola-R-CHP, Ven-R-CHOP, VR-CHOP, CR-CHOP) versus R-CHOP. Primary outcomes were PFS and OS, measured as HRs with 95% CIs. Secondary outcomes included grade 3–4 adverse events (AEs) from intention-to-treat (ITT) populations, as no study reported DEL-specific safety data.

All four efficacy studies defined DEL using the World Health Organization 2016 criteria (MYC immunohistochemistry ≥ 40% and BCL2 ≥ 50%, excluding cases with concurrent MYC and/or BCL2 gene rearrangements). Pathology review processes varied: POLARIX employed centralized pathology review with standardized IHC scoring; DEB used central review at designated study sites in China; CAVALLI used central pathology review at GOYA/CAVALLI study sites; and REMoDL-B used a combination of central and local pathology review, with our primary analysis restricted to IHC-confirmed DEL cases (*n* = 99).

### Data extraction and quality assessment

Two investigators independently extracted data using standardized forms. HRs were extracted directly from publications or estimated from Kaplan–Meier curves using the method of Tierney et al. (20). Risk of bias was assessed using the Cochrane RoB 2 tool for RCTs (21). Evidence quality was rated using the Grading of Recommendations, Assessment, Development, and Evaluations (GRADE) framework adapted for NMA.

### REMoDL-B DEL ascertainment and sensitivity analyses

For REMoDL-B, the primary analysis used 99 patients with IHC-confirmed DEL status (VR-CHOP, *n* = 45; R-CHOP, *n* = 54). As a sensitivity analysis, we developed a random forest prediction model (22) using gene expression data from the GSE117556 dataset to identify additional DEL patients, expanding the cohort to 125 patients (VR-CHOP, *n* = 58; R-CHOP, *n* = 67). The model was trained using 10-fold cross-validation with a probability threshold of 0.80 (positive predictive value > 90%). This strict threshold maximizes classification specificity but may systematically exclude borderline DEL cases with moderate MYC/BCL2 expression near the diagnostic cutoff; if these excluded patients had better outcomes than confirmed DEL cases (as suggested by the biological gradient of MYC/BCL2 expression), the observed VR-CHOP hazard ratio may represent a conservative estimate. A second sensitivity analysis completely excluded REMoDL-B (three studies only).

### Statistical analysis

We performed a frequentist random-effects NMA using the netmeta package in R (version 4.3.2) (23, 24). For the safety NMA, odds ratios (ORs) with 95% CIs were calculated from event counts in ITT populations. The network was star-shaped with no closed loops; therefore, consistency assessment via node-splitting was not feasible. Comparison-adjusted funnel plots were generated to evaluate small-study effects. Statistical significance was defined as *p* < 0.05 (two-sided). Multiple adverse events were compared across regimens in the safety analysis; therefore, a Bonferroni correction was additionally applied, using a family-wise threshold of *p* < 0.05/47 = 0.00106 across the 47 grade 3–4 AE comparisons. Both uncorrected and corrected results are reported for transparency (**Supplementary Table 3**).

As a supplementary analysis to address the inherent limitations of purely indirect comparisons in our star-shaped network, we performed a Bayesian NMA using two approaches. First, a fixed-effect Bayesian model with uninformative priors (d□ ~ Normal[0, 100^2^]) was used as the direct Bayesian counterpart to the primary frequentist analysis. Second, a random-effects Bayesian model with an informative prior on between-study heterogeneity (τ ~ Half-Normal(0, 0.32) based on empirically derived distributions for log-hazard ratios in oncology (25) was used to provide credible intervals that more fully capture structural uncertainty. Markov chain Monte Carlo sampling was performed with 200,000 iterations (50,000 burn-in; thinning every 15 iterations). Surface under the cumulative ranking curve (SUCRA) scores were computed from posterior rank probabilities. Additionally, we performed a prespecified sensitivity analysis excluding the CAVALLI study, which used a nonrandomized historical control from the GOYA trial, to assess whether our conclusions were robust to this methodological concern.

## Results

### Study selection and characteristics

**Figure 1:**
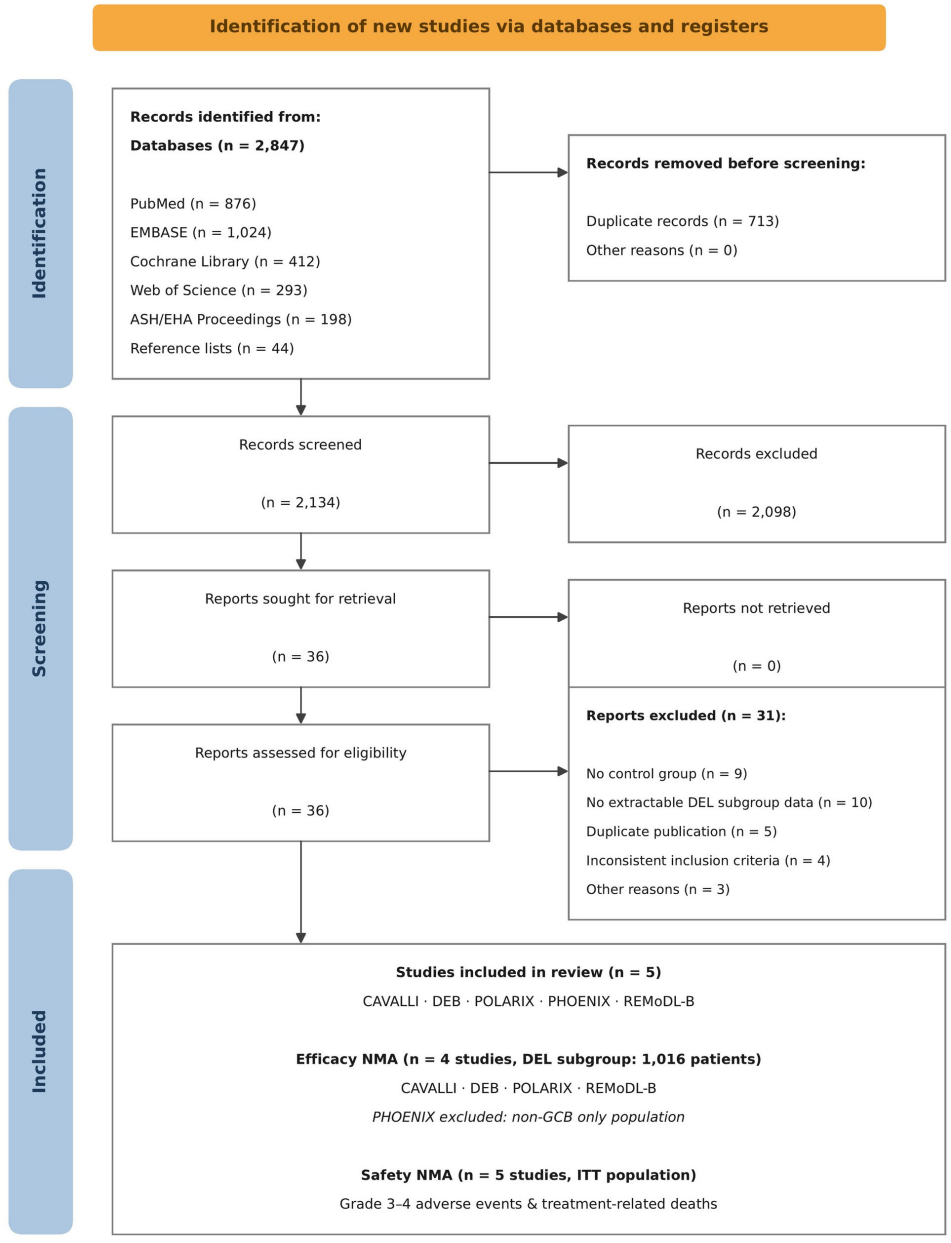
PRISMA flow diagram of study selection. A total of 2,847 records were identified through database searches. After duplicate removal and screening, four studies (CAVALLI, DEB, POLARIX, REMoDL-B) were included in the efficacy network meta-analysis, and five studies (additionally including PHOENIX) were included in the safety analysis.

**Figure 2:**
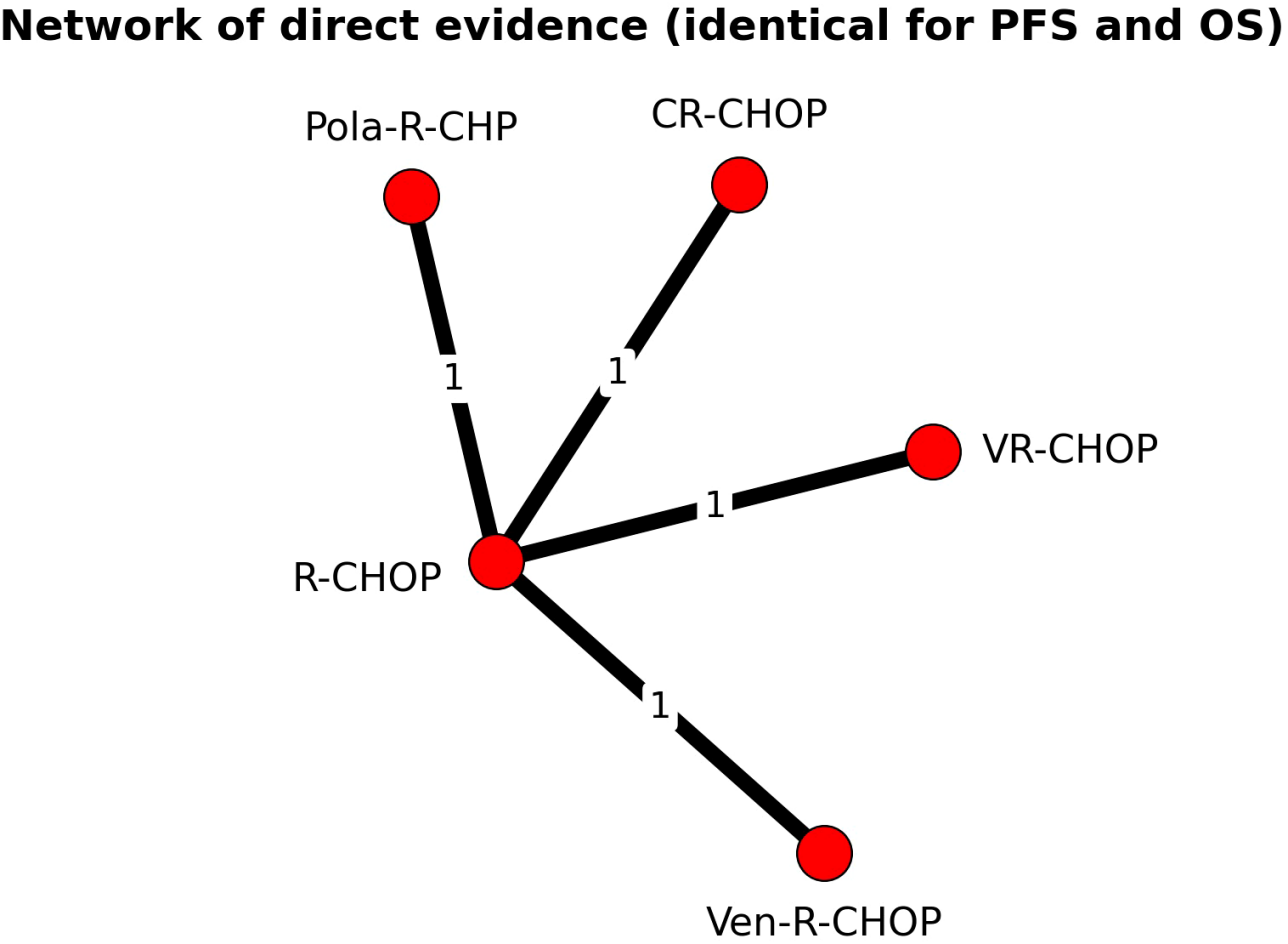
Network of direct evidence underlying the progression-free survival and overall survival analyses. Because the PFS and OS networks have identical geometry (the same four trials — POLARIX, DEB, REMoDL-B, CAVALLI — contributed to both outcomes), a single plot is presented. Each node represents a treatment regimen; lines connect treatments with direct comparisons, and the number on each line indicates the number of studies informing that comparison. R-CHOP serves as the common comparator in this star-shaped network.

The search identified 2,847 records; after removing 713 duplicates, 2,134 records were screened by title and abstract, and 36 underwent full-text review. Ultimately, four studies met the inclusion criteria for the efficacy NMA (**Figure 1**): CAVALLI (Ven-R-CHOP; single-arm phase 2 with GOYA historical controls), DEB (CR-CHOP; phase 3 RCT), POLARIX (Pola-R-CHP; phase 3 RCT), and REMoDL-B (VR-CHOP; phase 3 RCT). These four studies encompassed 1,016 DEL patients. PHOENIX was additionally included in the safety NMA (five studies total). The treatment network was star-shaped (**Figure 2**), with R-CHOP as the common comparator and each comparison based on a single study. Baseline characteristics are presented in **Table 1**.

**Table 1:** Baseline characteristics of included studies.

Characteristic	POLARIX		DEB		REMoDL-B		CAVALLI		PHOENIX^e^	
	Pola-R-CHP (*n* = 440)	R-CHOP (*n* = 439)	CR-CHOP (*n* = 211)^b^	R-CHOP (*n* = 212)^b^	VR-CHOP (*n* = 459)^c^	R-CHOP (*n* = 459)^c^	Ven-R-CHOP (*n* = 206)	R-CHOP (*n* = 564)^d^	Ibr-R-CHOP (*n* = 419)	R-CHOP (*n* = 419)
Study design	Phase III RCT		Phase III RCT		Phase III RCT		Phase II + historical control		Phase III RCT	
DEL subgroup	139	151	211	212	45	54	80	124	Not included	Not included
Age (median; range/IQR; year)	65 (19–80)	66 (19–80)	64 (IQR 55–68)^f^	62 (IQR 54–67)^f^	63 (20–84)	65 (24–86)	65 (18–85)	62 (18–83)	63 (19–88)	61 (19–87)
Male (*n*; %)	239 (54.3)	234 (53.3)	91 (43.1)	110 (51.9)	NR	NR	113 (54.9)	297 (52.7)	221 (52.7)	226 (55.6)
Ann Arbor III–IV (*n*; %)	393 (89.3)	387 (88.2)	170 (80.6)	174 (82.1)	317 (69.4)	314 (68.7)	NA	NA	318 (75.9)	315 (75.2)
IPI 3–5 (*n*; %)	273 (62.0)	272 (62.0)	116 (55.0)	121 (57.1)	216 (47.1)	225 (49.0)	NA	NA	183 (43.7)	181 (43.2)
ECOG PS 0–1 (*n*; %)	374 (85.0)	363 (82.7)	160 (75.8)	160 (75.5)	390 (88.0)	392 (88.5)	172 (84)	476 (84)	381 (90.9)	357 (85.2)
ECOG PS ≥ 2 (*n*; %)	66 (15.0)	75 (17.1)	51 (24.2)	52 (24.5)	53 (12.0)	51 (11.5)	34 (17)	87 (15)	38 (9.1)	62 (14.8)
LDH elevated (*n*; %)	291 (66.1)	284 (64.7)	137 (64.9)	147 (69.3)	227 (61.7)	224 (59.4)	121 (58.7)	322 (57.1)	234 (55.8)	220 (52.5)
Extranodal sites ≥ 2 (*n*; %)	213 (48.4)	213 (48.5)	40 (19.0)	42 (19.8)	NR	NR	140 (68.0)	370 (65.6)	31.0% (≥ 1)^g^	37.2% (≥ 1)^g^
Bulky disease ≥ 7.5 cm (*n*; %)	193 (43.9)	192 (43.7)	43 (20.4)	48 (22.6)	141 (31.3)^j^	122 (26.8)^j^	NR	209 (37.1)	14.3% (≥ 10 cm)^g^	14.1% (≥ 10 cm)^g^
GCB subtype (*n*; %)	187 (55.5)	170 (48.3)	45 (21.3)	61 (28.8)	235 (51.2)	240 (52.3)	101 (59)	213 (57)	0 (0)^h^	0 (0)^h^
ABC subtype (*n*; %)	106 (31.5)	129 (36.6)	NA	NA	123 (26.8)	121 (26.4)	48 (28)	104 (28)	285 (77.0)^h^	282 (74.8)^h^
Double-expressor (*n*)^k^	139^a^	151^a^	211	212	45	54	80	124	386	380
MYC/BCL2 rearrangement (DHL/THL) (*n*; %)^i^	26 (7.9)	19 (5.7)	4 (1.9)	7 (3.3)	17/172 (9.9)	18/188 (9.6)	7 (5)	8 (3)	Rare	Rare
MYC rearrangement (*n*; %)^i^	NR	NR	NA	NA	24/172 (14.0)	27/188 (14.4)	NR	NR	NR	NR
Genetic subtype (LymphGen) (*n*)^l^	Performed (*n* NR)^l^		Not performed		Not performed		Not performed		MCD 110/BN2 47/N1 28 (pooled, 203 classified)^l^	
Median follow-up (months)	64.1	64.1	41.3	41.3	30	30	32.4	32.4	34.8	34.8

Data are from ITT or total study populations unless otherwise noted. Percentages are calculated from patients with available data. Baseline clinical and demographic characteristics of the ITT populations and DEL subgroups across included trials (POLARIX, DEB, REMoDL-B, CAVALLI, PHOENIX). Data include age, gender distribution, Ann Arbor stage, IPI score, ECOG PS, laboratory and disease-related features (LDH elevation, extranodal sites, bulky disease), cell-of-origin subtype, DEL prevalence, and median follow-up duration. Subgroup-specific annotations denote trial design nuances, DEL ascertainment methods, and population exclusions (e.g., PHOENIX exclusively enrolled nongerminal center B-cell [non-GCB] patients).

ABC, activated B-cell; CR-CHOP, chidamide + R-CHOP; DEL, double-expressor lymphoma; ECOG PS, Eastern Cooperative Oncology Group performance status; GCB, germinal center B-cell; Ibr-R-CHOP, ibrutinib + R-CHOP; IHC, immunohistochemistry; IPI, International Prognostic Index; IPD, individual patient data; IQR, interquartile range; ITT, intention-to-treat; LDH, lactate dehydrogenase; NA, not applicable; NMA, network meta-analysis; NR, not reported; Pola-R-CHP, polatuzumab vedotin + R-CHP; RCT, randomized controlled trial; R-CHOP, rituximab–cyclophosphamide–doxorubicin–vincristine–prednisone; RF, random forest; Ven-R-CHOP, venetoclax + R-CHOP; VR-CHOP, bortezomib + R-CHOP.

**Figure 3:**
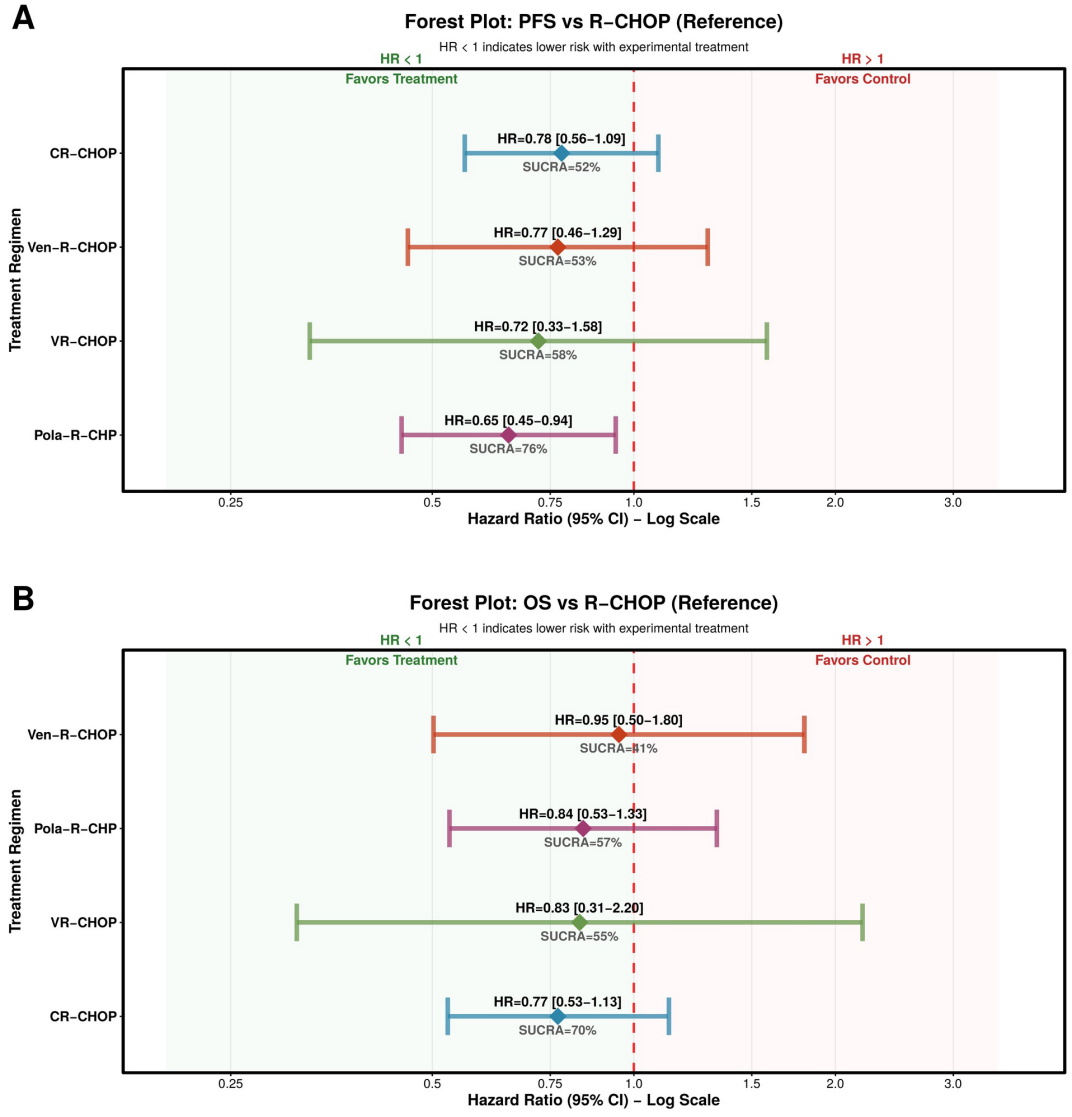
Forest plots of network meta-analysis results vs. R-CHOP. **(A)** Progression-free survival. **(B)** Overall survival. Hazard ratios (HRs) with 95% confidence intervals (CIs) are shown. HR < 1 favors the experimental regimen over R-CHOP. The dashed vertical line indicates HR = 1 (no difference). SUCRA, surface under the cumulative ranking curve.

^a^ POLARIX: double-expressor lymphoma (IHC) subgroup, global population; subgroup counts and PFS/OS data are from the 5-year analysis [see **Figure 3** of (13)]; baseline ITT characteristics as from Tilly et al. (12).

^b^ DEB: the entire cohort consisted of patients with DEL (100%); prospective, randomized, double-blind, placebo-controlled phase 3 trial (14).

^c^ REMoDL-B: an IHC-defined DEL cohort was reconstructed from the publicly available individual-level data (Gene Expression Omnibus, GSE117556) by applying the WHO 2016 IHC thresholds (MYC ≥40%, BCL2 ≥50%) and restricting to IPI ≥2 to preserve transitivity with the IPI ≥2 comparisons in the network (notably the CAVALLI/GOYA comparison); this yielded 99 patients (VR-CHOP, n=45; R-CHOP, n=54), from which the hazard ratios were derived by the authors.

^d^ CAVALLI: single-arm Ven-R-CHOP (*n* = 206) was compared with a historical R-CHOP control from the GOYA trial (*n* = 564, IPI ≥ 2) (13).

^e^ PHOENIX: included in the safety NMA only (no DEL-specific PFS/OS data available); non-GCB subtype selected (Younes et al. Lancet Oncol. 2019;20:849–861).

^f^ DEB: age is reported as median (IQR) rather than median (range).

^g^ PHOENIX: extranodal sites are reported as ≥ 1 (not ≥ 2); bulky disease is defined as ≥ 10 cm (not ≥ 7.5 cm).

^h^ PHOENIX: enrolled only non-GCB patients; GCB = 0% by design.

^i^ MYC/BCL2 rearrangement: double-expressor lymphoma (DEL) is defined by MYC/BCL2 protein coexpression in the absence of MYC/BCL2 (± BCL6) rearrangements (WHO 2016); double-hit/triple-hit (DHL/THL) cases were excluded from the DEL efficacy subgroups in POLARIX, REMoDL-B, and CAVALLI; DEB was analyzed in all randomized patients by intention-to-treat (see DEB note below). POLARIX: 26/331 vs. 19/334 by central FISH (12). DEB: MYC/BCL2 (± BCL6) rearrangements were identified in four (CR-CHOP) and seven (R-CHOP) patients (11 total, 2.6%); per the trial’s intention-to-treat design, these patients were retained in the analyzed 211/212 cohort on which the reported HRs are based (14). CAVALLI: BCL-2 and MYC FISH-positive 7/139 (Ven-R-CHOP) vs. 8/230 (GOYA R-CHOP). REMoDL-B: 17/172 (RB-CHOP) vs. 18/188 (R-CHOP) DHL among FISH-evaluable cases (15), excluded from the IHC-defined DEL subgroups. PHOENIX: enrolled non-GCB patients only, in whom DHL is rare/effectively absent. Rates are computed within each trial’s own FISH/rearrangement-evaluable subset; denominators differ (POLARIX 331/334; CAVALLI 139/230; REMoDL-B 172/188) and are not directly comparable.

^j^ REMoDL-B: bulky disease is defined as > 10 cm (vs. the ≥ 7.5-cm threshold used elsewhere) (15).

^k^ Values are the double-expressor (DEL) subgroup counts analyzed in each trial. DEL was ascertained within each trial’s own population (all-DEL cohort in DEB, biomarker-enriched cohort in CAVALLI, and biomarker-evaluable subsets in POLARIX and REMoDL-B; the matched GOYA R-CHOP arm as CAVALLI’s control), so prevalence proportions are not directly comparable and are not tabulated. The analyzed DEL subgroups appear in the DEL subgroup row and in **Table 2**.

^l^ Genetic (LymphGen) subtyping: PHOENIX—MCD 110, BN2 47, N1–28 of 203 classified (773 sequenced; 18 composite excluded; A53 not assessable), both arms pooled (Wilson WH, et al. Cancer Cell 2021;39:1643–1653). POLARIX—LymphGen was performed on 594 WES-evaluable patients; per-subtype prevalence was reported only graphically and not extractable (Morschhauser et al. Blood 2023;142:3000). LymphGen subtyping was not performed in DEB, REMoDL-B, or CAVALLI.

### Progression-free survival

The NMA for PFS (**Figure 3A**; **Table 2**) demonstrated that Pola-R-CHP was the only regimen achieving a statistically significant improvement versus R-CHOP (HR = 0.65, 95% CI = 0.45–0.94, *p* = 0.02; SUCRA = 76.1%). VR-CHOP (HR = 0.72, 95% CI = 0.33–1.58; SUCRA = 58.3%), Ven-R-CHOP (HR = 0.77, 95% CI = 0.46–1.29; SUCRA = 52.5%), and CR-CHOP (HR = 0.78, 95% CI = 0.56–1.09; SUCRA = 51.9%) showed numerically favorable but nonsignificant trends. Notably, VR-CHOP, Ven-R-CHOP, and CR-CHOP had closely overlapping SUCRA values (51.9%–58.3%), and these differences should not be interpreted as meaningful distinctions. Only Pola-R-CHP achieved statistical significance; SUCRA rankings for the remaining regimens should be considered auxiliary references rather than definitive conclusions. SUCRA represents a ranking probability and does not denote statistical significance.

**Table 2:** Network meta-analysis results: efficacy outcomes (DEL subgroup, IHC gold standard).

Treatment	HR (95% CI)	p-value	SUCRA (%)
Progression-free survival (PFS)			
Pola-R-CHP	0.65 (0.45–0.94)*	0.0219	76.1%
VR-CHOP	0.72 (0.33–1.58)	0.4156	58.3%
Ven-R-CHOP	0.77 (0.46–1.29)	0.3204	52.5%
CR-CHOP	0.78 (0.56–1.09)	0.1436	51.9%
R-CHOP	Reference	–	11.2%
Overall survival (OS)			
CR-CHOP	0.77 (0.53–1.13)	0.1760	69.8%
Pola-R-CHP	0.84 (0.53–1.33)	0.4576	56.8%
VR-CHOP	0.83 (0.31–2.20)	0.7122	54.7%
Ven-R-CHOP	0.95 (0.50–1.80)	0.8747	41.0%
R-CHOP	Reference	–	27.7%

HR < 1 favors experimental treatment over R-CHOP. Higher SUCRA values indicate better ranking probability. REMoDL-B data based on the immunohistochemistry (IHC) gold standard (*n* = 45 VR-CHOP vs. *n* = 54 R-CHOP). Total efficacy NMA population: 1,016 DEL patients across four studies. Frequentist network meta-analysis (NMA) results for progression-free survival (PFS) and overall survival (OS) in the DEL subgroup (defined by WHO 2016 IHC gold standard: MYC ≥ 40% and BCL2 ≥ 50%, no gene rearrangements). Data include hazard ratios (HRs) with 95% confidence intervals (CIs), two-sided *p*-values, and surface under the cumulative ranking curve (SUCRA) scores for each novel R-CHOP-based regimen vs. standard R-CHOP. Higher SUCRA values indicate a higher probability of superior treatment ranking; statistically significant HRs (*p* < 0.05) are marked with an asterisk. Analyses are based on 1,016 DEL patients across four studies, with REMoDL-B data restricted to IHC-confirmed DEL cases.

HR, hazard ratio; CI, confidence interval; SUCRA, surface under the cumulative ranking curve.

^*^p < 0.05, statistically significant. Bold values indicate statistically significant results (*p* < 0.05).

### Overall survival

**Figure 4:**
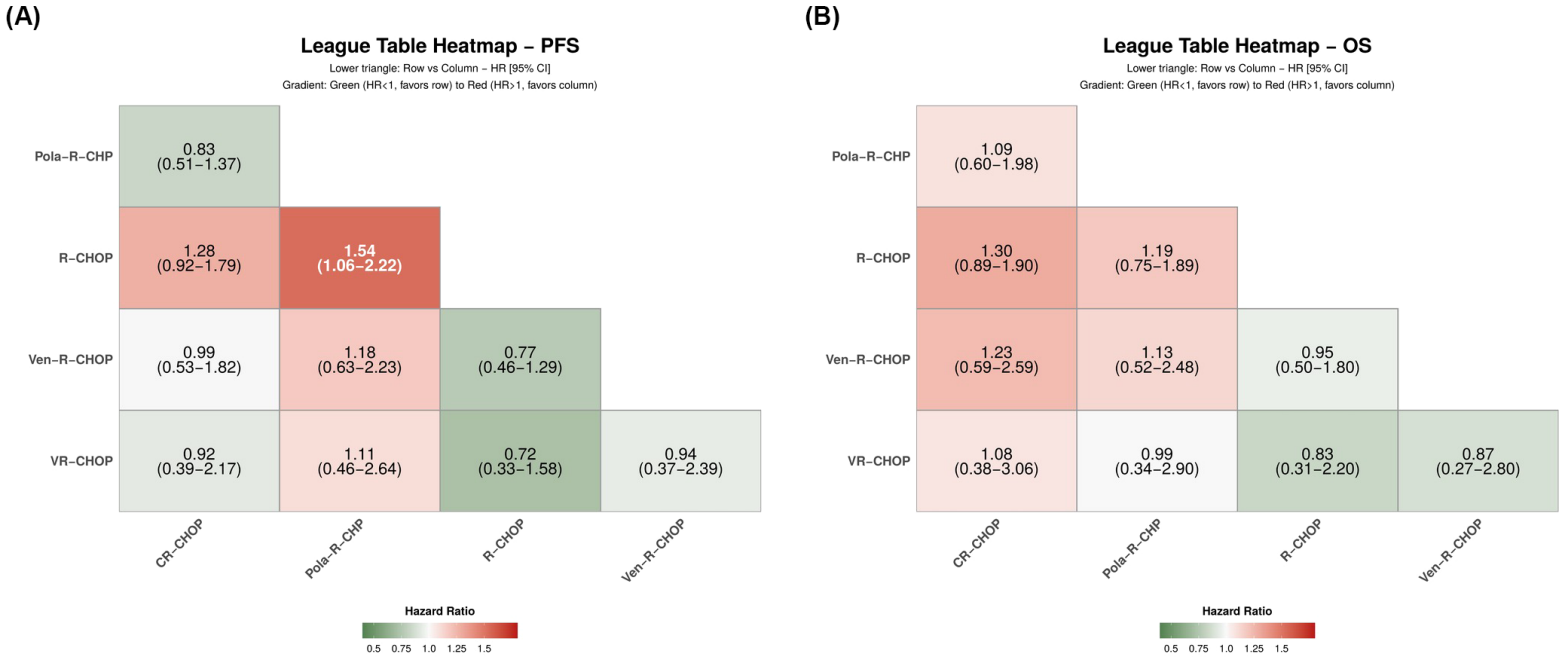
League table heatmaps of pairwise comparisons. **(A)** PFS. **(B)** OS. Each cell shows the HR (95% CI) for the row treatment vs. the column treatment. HR < 1 favors the row treatment. Statistically significant results (*p* < 0.05) are highlighted.

No regimen significantly improved OS versus R-CHOP (all *p* > 0.05; **Figure 3B**). CR-CHOP showed the most favorable OS trend (HR = 0.77, 95% CI = 0.53–1.13; SUCRA = 69.8%), followed by Pola-R-CHP (HR = 0.84, 95% CI = 0.53–1.33; SUCRA = 56.8%) and VR-CHOP (HR = 0.83, 95% CI = 0.31–2.20; SUCRA = 54.7%). Ven-R-CHOP showed the least OS benefit (HR = 0.95, 95% CI = 0.50–1.80; SUCRA = 41.0%). The league table of pairwise comparisons (**Figure 4**) demonstrated that R-CHOP was significantly inferior to Pola-R-CHP for PFS (HR = 1.54, 95% CI = 1.06–2.22), but no significant differences were observed for OS between any pair.

### Safety

**Figure 5:**
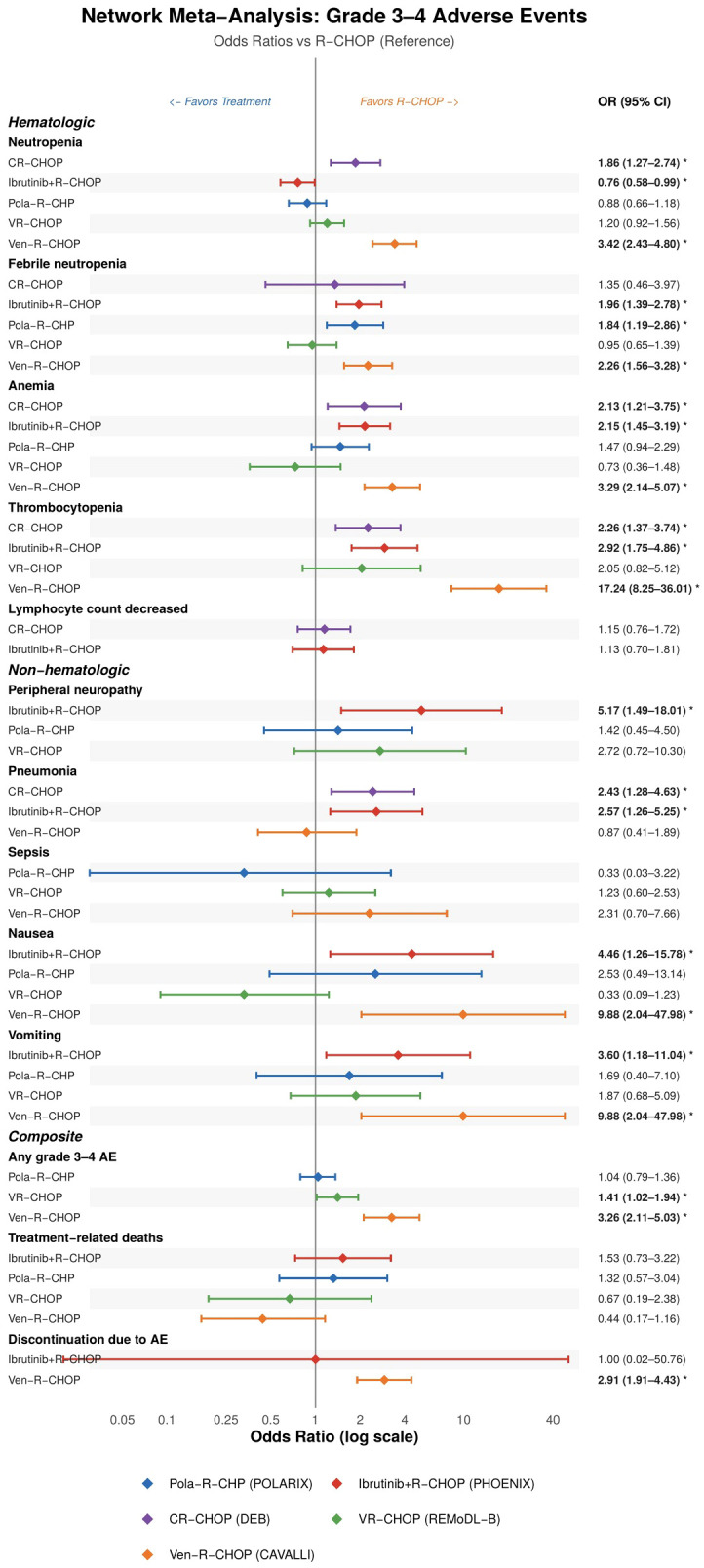
Forest plot of grade 3–4 adverse events. Odds ratios (ORs) with 95% CIs for each treatment regimen vs. R-CHOP are shown for individual adverse events and for any grade 3–4 adverse event. OR > 1 indicates a higher risk with the experimental treatment. Safety data were derived from intention-to-treat populations. Asterisks indicate nominal statistical significance (uncorrected *p* < 0.05); Bonferroni-adjusted results are provided in **Supplementary Table 3**.

Safety data were derived from ITT/safety-evaluable populations, as no included trial reported DEL-specific adverse event data; these safety ORs therefore reflect overall trial populations and may not precisely represent the toxicity experience of the DEL subgroup, who typically present with higher IPI scores, more advanced stage, and potentially different bone marrow reserves. To account for multiple testing, a Bonferroni-corrected threshold (*p* < 0.05/47 = 0.00106) was applied across all safety comparisons (**Supplementary Table 3**); comparisons that did not meet this threshold are interpreted as nominal and hypothesis-generating. With this caveat, the safety NMA (**Table 3**; **Figure 5**) revealed distinct toxicity profiles. Ven-R-CHOP was associated with significantly increased risks of grade 3–4 thrombocytopenia (OR = 17.24, 95% CI = 8.25–36.01), neutropenia (OR = 3.42, 95% CI = 2.43–4.80), anemia (OR = 3.29, 95% CI = 2.14–5.07), febrile neutropenia (OR = 2.26, 95% CI = 1.56–3.28), any grade 3–4 AE (OR = 3.26, 95% CI = 2.11–5.03), and treatment discontinuation due to adverse events (OR = 2.91, 95% CI = 1.91–4.43), all of which retained significance after Bonferroni correction. The extreme magnitude of the Ven-R-CHOP thrombocytopenia OR should be interpreted with particular caution, as it may be partially attributable to systematic differences in adverse event ascertainment and reporting standards between the prospective CAVALLI cohort and the retrospective GOYA historical control arm, and it should not be regarded as confirmatory evidence of toxicity. CR-CHOP showed nominally increased neutropenia (OR = 1.86), anemia (OR = 2.13), thrombocytopenia (OR = 2.26), and pneumonia (OR = 2.43) risks, none of which retained significance after Bonferroni correction. VR-CHOP nominally increased the overall grade 3–4 AE risk (OR = 1.41, 95% CI = 1.02–1.94), which likewise did not retain significance after correction. Pola-R-CHP demonstrated a safety profile comparable to R-CHOP (any grade 3–4 AE: OR = 1.04, 95% CI = 0.79–1.36). Treatment-related mortality did not differ significantly for any regimen. The PHOENIX study contributed safety data showing that ibrutinib plus R-CHOP was associated with increased peripheral neuropathy (OR = 5.17), nausea (OR = 4.46), and vomiting (OR = 3.60); these neurologic and gastrointestinal signals were nominal and did not retain significance after Bonferroni correction, whereas its hematologic toxicity (febrile neutropenia, anemia, and thrombocytopenia) remained significant after correction.

**Table 3:** Frequentist NMA results for grade 3–4 adverse events vs. R-CHOP (safety-evaluable/treated populations).

Adverse event	Regimen	OR (95% CI)	*p*-value	Experimental (*n*/*N*; %)	R-CHOP (*n*/*N*; %)
Hematologic					
Neutropenia	CR-CHOP	1.86 (1.27–2.74)^*^	0.0016	128/211 (60.7)	96/212 (45.3)
	Pola-R-CHP	0.88 (0.66–1.18)	0.3858	123/435 (28.3)	135/438 (30.8)
	VR-CHOP	1.20 (0.92–1.56)	0.1708	219/444 (49.3)	200/447 (44.7)
	Ven-R-CHOP	3.42 (2.43–4.80)^*,**^	< 0.001	141/206 (68.4)	219/564 (38.8)
	Ibrutinib + R-CHOP	0.76 (0.58–0.99)^*^	0.0446	212/416 (51.0)	242/418 (57.9)
Febrile neutropenia	CR-CHOP	1.35 (0.46–3.97)	0.5800	8/211 (3.8)	6/212 (2.8)
	Pola-R-CHP	1.84 (1.19–2.86)^*,a^	0.0070	60/435 (13.8)	35/438 (8.0)
	VR-CHOP	0.95 (0.65–1.39)	0.8017	60/444 (13.5)	63/447 (14.1)
	Ven-R-CHOP	2.26 (1.56–3.28)^*,**^	< 0.001	63/206 (30.6)	92/564 (16.3)
	Ibrutinib + R-CHOP	1.96 (1.39–2.78)^*,**^	0.0001	106/416 (25.5)	62/418 (14.8)
Anemia	CR-CHOP	2.13 (1.21–3.75)^*^	0.0091	40/211 (19.0)	21/212 (9.9)
	Pola-R-CHP	1.47 (0.94–2.29)	0.0883	52/435 (12.0)	37/438 (8.4)
	VR-CHOP	0.73 (0.36–1.48)	0.3875	14/444 (3.2)	19/447 (4.3)
	Ven-R-CHOP	3.29 (2.14–5.07)^*,**^	< 0.001	50/206 (24.3)	50/564 (8.9)
	Ibrutinib + R-CHOP	2.15 (1.45–3.19)^*,**^	0.0001	84/416 (20.2)	44/418 (10.5)
Thrombocytopenia	CR-CHOP	2.26 (1.37–3.74)^*^	0.0015	54/211 (25.6)	28/212 (13.2)
	VR-CHOP	2.05 (0.82–5.12)	0.1258	14/444 (3.2)	7/447 (1.6)
	Ven-R-CHOP	17.24 (8.25–36.01)^*,**^	< 0.001	45/206 (21.8)	9/564 (1.6)
	Ibrutinib + R-CHOP	2.92 (1.75–4.86)^*,**^	< 0.001	58/416 (13.9)	22/418 (5.3)
Lymphocyte count decreased	CR-CHOP	1.15 (0.76–1.72)	0.5100	72/211 (34.1)	66/212 (31.1)
	Ibrutinib + R-CHOP	1.13 (0.70–1.81)	0.6150	40/416 (9.6)	36/418 (8.6)
Nonhematologic					
Peripheral neuropathy	Pola-R-CHP	1.42 (0.45–4.50)^a^	0.5602	7/435 (1.6)	5/438 (1.1)
	VR-CHOP	2.72 (0.72–10.30)	0.1420	8/444 (1.8)	3/447 (0.7)
	Ibrutinib + R-CHOP	5.17 (1.49–18.01)^*^	0.0098	15/416 (3.6)	3/418 (0.7)
Pneumonia	CR-CHOP	2.43 (1.28–4.63)^*^	0.0066	33/211 (15.6)	15/212 (7.1)
	Ven-R-CHOP	0.87 (0.41–1.89)	0.7325	9/206 (4.4)	28/564 (5.0)
	Ibrutinib + R-CHOP	2.57 (1.26–5.25)^*^	0.0097	27/416 (6.5)	11/418 (2.6)
Sepsis	Pola-R-CHP	0.33 (0.03–3.22)	0.3412	1/435 (0.2)	3/438 (0.7)
	VR-CHOP	1.23 (0.60–2.53)	0.5710	17/444 (3.8)	14/447 (3.1)
	Ven-R-CHOP	2.31 (0.70–7.66)	0.1699	5/206 (2.4)	6/564 (1.1)
Nausea	Pola-R-CHP	2.53 (0.49–13.14)^a^	0.2702	5/435 (1.1)	2/438 (0.5)
	VR-CHOP	0.33 (0.09–1.23)	0.0990	3/444 (0.7)	9/447 (2.0)
	Ven-R-CHOP	9.88 (2.04–47.98)^*^	0.0045	7/206 (3.4)	2/564 (0.4)
	Ibrutinib + R-CHOP	4.46 (1.26–15.78)^*^	0.0203	13/416 (3.1)	3/418 (0.7)
Vomiting	Pola-R-CHP	1.69 (0.40–7.10)^a^	0.4803	5/435 (1.1)	3/438 (0.7)
	VR-CHOP	1.87 (0.68–5.09)	0.2226	11/444 (2.5)	6/447 (1.3)
	Ven-R-CHOP	9.88 (2.04–47.98)^*^	0.0045	7/206 (3.4)	2/564 (0.4)
	Ibrutinib + R-CHOP	3.60 (1.18–11.04)^*^	0.0248	14/416 (3.4)	4/418 (1.0)
Composite and treatment outcomes					
Any grade 3–4 AE	Pola-R-CHP	1.04 (0.79–1.36)^a^	0.8578	264/435 (60.7)	262/438 (59.8)
	VR-CHOP	1.41 (1.02–1.94)^*^	0.0385	362/444 (81.5)	339/447 (75.8)
	Ven-R-CHOP	3.26 (2.11–5.03)^*,**^	< 0.001	178/206 (86.4)	373/564 (66.1)
Treatment-related deaths	Pola-R-CHP	1.32 (0.57–3.04)^a^	0.5237	13/435 (3.0)	10/438 (2.3)
	VR-CHOP	0.67 (0.19–2.38)	0.5344	4/444 (0.9)	6/447 (1.3)
	Ven-R-CHOP	0.44 (0.17–1.16)	0.0965	5/206 (2.4)	30/564 (5.3)
	Ibrutinib + R-CHOP	1.53 (0.73–3.22)	0.2621	18/416 (4.3)	12/418 (2.9)
Discontinuation due to AE	Ven-R-CHOP	2.91 (1.91–4.43)^*,**^	< 0.001	50/206 (24.3)	56/564 (9.9)
	Ibrutinib + R-CHOP	1.00 (0.02–50.76)^b^	0.9981	0/416 (0.0)	0/418 (0.0)

OR > 1 indicates a higher risk than the in-trial R-CHOP comparator. Safety data are derived from each trial’s safety-evaluable/treated population (no DEL-specific data were available); the POLARIX denominators (435/438) therefore differ from the ITT denominators in **Table 1** (440/439). Ibrutinib + R-CHOP data are from PHOENIX (safety only; excluded from the efficacy NMA). Frequentist NMA results for grade 3–4 AEs in the ITT populations of included trials (POLARIX, DEB, REMoDL-B, CAVALLI, PHOENIX). Data include ORs with 95% CIs, two-sided *p*-values, event counts in treatment and control arms, and absolute event rates for hematologic (neutropenia, febrile neutropenia, anemia, thrombocytopenia) and nonhematologic (peripheral neuropathy, pneumonia, sepsis, nausea, vomiting) AEs, as well as composite any grade 3–4 AE and treatment-related mortality. OR > 1 indicates a higher risk of the AE with the experimental regimen vs. R-CHOP; statistically significant results (*p* < 0.05) are marked with an asterisk. Safety data are derived from ITT populations (no DEL-specific AE data were reported in included trials).

AE, adverse event; CI, confidence interval; CR-CHOP, chidamide + R-CHOP; NMA, network meta-analysis; OR, odds ratio; Pola-R-CHP, polatuzumab vedotin + R-CHP; R-CHOP, rituximab–cyclophosphamide–doxorubicin–vincristine–prednisone; Ven-R-CHOP, venetoclax + R-CHOP; VR-CHOP, bortezomib + R-CHOP.

^*^ Nominal *p* < 0.05; ^**^p < 0.05/47 = 0.00106 (retains significance after Bonferroni correction).

^a^ Recomputed on the corrected POLARIX safety-evaluable population (435/438), change vs. prior estimate ≤ 0.01.

^b^ Zero events in both arms (OR not reliably estimable).

### Bias assessment and sensitivity analyses

Each comparison was based on a single study, so between-study heterogeneity could not be statistically estimated, and consistency testing via node-splitting was infeasible in the star-shaped network. Comparison-adjusted funnel plots (**Supplementary Figure 1**) showed a symmetric distribution without evidence of publication bias. GRADE assessment (**Table 4**) rated the Pola-R-CHP versus R-CHOP PFS evidence as moderate quality (downgraded for indirectness) and OS evidence as low quality (downgraded for imprecision and indirectness). A supplementary Bayesian NMA (**Supplementary Table 2**) yielded results highly concordant with the primary frequentist analysis. Under the fixed-effect Bayesian model with uninformative priors, point estimates and credible intervals were virtually identical to frequentist confidence intervals. Under the random-effects model with an informative Turner et al. (25) prior, credible intervals were appropriately wider (e.g., Pola-R-CHP PFS: 95% CI = 0.30–1.45 vs. frequentist: 95% CI = 0.45–0.94), reflecting additional structural uncertainty in the sparse star-shaped network. The posterior probability that Pola-R-CHP improves PFS relative to R-CHOP was 90.2%. SUCRA rankings were concordant across frameworks: Pola-R-CHP ranked first for PFS in the frequentist (76.1%), Bayesian fixed-effect (76.2%), and Bayesian random-effects (69.7%) analyses. A sensitivity analysis excluding CAVALLI (which used nonrandomized historical controls from GOYA) preserved the primary findings: with three remaining studies (POLARIX, DEB, REMoDL-B), Pola-R-CHP maintained a statistically significant PFS benefit (HR = 0.65, 95% CI = 0.45–0.94; SUCRA = 78.2%), and SUCRA rankings for the remaining treatments were stable. The Bayesian random-effects analysis confirmed robustness (Pola-R-CHP PFS posterior P[HR < 1] = 90.3%). Two additional sensitivity analyses confirmed robustness: expanding the REMoDL-B DEL cohort to 125 patients using the prediction model yielded consistent rankings (Pola-R-CHP PFS HR = 0.65, SUCRA = 78.1%), and excluding REMoDL-B entirely (three studies) maintained the significance of Pola-R-CHP’s PFS benefit (HR = 0.65, 95% CI = 0.45–0.94).

**Table 4:** Grading of recommendations, assessment, development, and evaluations (GRADE) framework assessment of evidence quality for each novel R-CHOP-based regimen vs. R-CHOP for PFS in the DEL subgroup.

Comparison (treatment vs. R-CHOP)	No. of studies	Design	Risk of bias	Inconsistency	Indirectness	Imprecision	Other considerations	HR (95% CI)	SUCRA (%)	Overall quality	Importance
Pola-R-CHP vs. R-CHOP	1 (POLARIX)	RCT subgroup	Not seriousa	N/A (single study)	Seriousb	Not serious	None	0.65 (0.45–0.94)*	76.1	⊕⊕⊕○: moderate	Critical
CR-CHOP vs. R-CHOP	1 (DEB)	RCT	Not serious	N/A (single study)	Seriousc	Seriousd	None	0.78 (0.56–1.09)	51.9	⊕⊕○○: low	Critical
VR-CHOP vs. R-CHOP	1 (REMoDL-B)	RCT subgroup	Not serious	N/A (single study)	Seriousb	Very seriouse	None	0.72 (0.33–1.58)	58.3	⊕○○○: very low	Important
Ven-R-CHOP vs. R-CHOP	1 (CAVALLI)	Single-arm + historical control	Seriousf	N/A (single study)	Seriousb	Seriousd	None	0.77 (0.46–1.29)	52.5	⊕○○○: very low	Important

Starting level: RCT = high; observational/historical control = low. Downgraded for risk of bias, inconsistency, indirectness, and imprecision. Ratings are based on study design, risk of bias, inconsistency, indirectness, imprecision, and other considerations, with evidence quality categorized as high (⊕⊕⊕⊕), moderate (⊕⊕⊕○), low (⊕⊕○○), or very low (⊕○○○). The table includes the number of studies, HR (95% CI), SUCRA score, overall evidence quality, and clinical importance for each comparison, with footnotes detailing the rationale for quality downgrades (e.g., indirectness from subgroup analyses, imprecision from wide CIs, serious risk of bias from historical control design). Consistency assessment was not applicable (N/A) due to the star-shaped network structure.

*N/A*, not applicable (star-shaped network precludes consistency assessment between non-R-CHOP comparisons).

^*^ *p* < 0.05, statistically significant.

^a^ POLARIX: low risk of bias by RoB 2.0 for the parent trial; subgroup analysis was prespecified.

^b^ Indirectness: efficacy data derived from DEL subgroup analyses of larger trials, not dedicated DEL trials. The population may not be fully representative.

^c^ Indirectness: DEB enrolled 100% DEL patients (direct evidence), but a 100% Chinese population limits generalizability.

^d^ Imprecision: 95% CI crosses 1.0; optimal information size not met.

^e^ Very serious imprecision: wide CI (0.33–1.58) with a small DEL subgroup in REMoDL-B.

^f^ CAVALLI: single-arm phase II study with a historical control from the GOYA trial; high risk of selection bias, no randomization for the Ven-R-CHOP arm.

## Discussion

To our knowledge, this is the first NMA specifically comparing R-CHOP-based regimens in DEL patients. The principal finding is that Pola-R-CHP is the only regimen demonstrating statistically significant PFS improvement (HR = 0.65, 35% risk reduction), with a safety profile comparable to R-CHOP. This finding aligns with the recently reported 5-year POLARIX follow-up showing sustained PFS benefit in the DEL subgroup (11).

The PFS advantage of Pola-R-CHP is mechanistically plausible. Polatuzumab vedotin, an anti-CD79b antibody–drug conjugate, delivers the microtubule inhibitor MMAE directly to B cells via receptor-mediated internalization (26, 27). This targeted cytotoxic mechanism may be particularly effective against DEL tumor cells that are resistant to conventional chemotherapy due to BCL2-mediated apoptosis evasion, as polatuzumab vedotin bypasses the mitochondrial apoptotic pathway.

A notable finding is the dissociation between PFS and OS outcomes. Despite Pola-R-CHP’s significant PFS improvement, no regimen achieved a statistically significant OS benefit. This pattern is consistent with the 5-year POLARIX data showing a maintained PFS benefit but nonsignificant OS differences in the DEL subgroup (11). Several factors may explain this dissociation. First, the availability of effective postprogression therapies, particularly chimeric antigen receptor T-cell (CAR T) therapy and bispecific antibodies (28), creates a dilution effect on OS by rescuing patients who relapse after first-line treatment. Second, OS analyses may be underpowered given the relatively small sample sizes and immature follow-up in the DEL subgroups. Whether PFS represents a valid surrogate endpoint for DEL patients requires further validation with longer follow-up (29). Notably, the relative treatment rankings for OS were not concordant with those observed for PFS, and the regimen achieving the highest OS ranking did not reach statistical significance. These discordant OS findings, likely attributable to the smaller number of OS events, immature follow-up, and the confounding influence of effective postrelapse salvage therapy, should be regarded as hypothesis-generating rather than confirmatory and require validation in prospective trials with longer follow-up.

The safety analysis raises important considerations, though these results must be interpreted in light of their derivation from ITT populations rather than DEL subgroups specifically. Ven-R-CHOP was associated with substantial hematologic toxicity, including a 17-fold increased thrombocytopenia risk. However, this extreme OR should be interpreted cautiously: the CAVALLI study used historical controls from the GOYA trial, and systematic differences in AE reporting standards between historical controls and prospective single-arm studies (e.g., reporting thresholds, monitoring frequency, and attribution criteria) may partially account for this effect magnitude (13). This extreme estimate should therefore not be regarded as confirmatory evidence of toxicity, and a prespecified sensitivity analysis excluding CAVALLI left the regimen ranking and principal conclusions unchanged. Conversely, Pola-R-CHP demonstrated remarkable safety with no increase in overall grade 3–4 AEs (OR = 1.04), supporting its favorable benefit–risk profile. Ibrutinib plus R-CHOP (PHOENIX) showed increased peripheral neuropathy and gastrointestinal toxicity (nominal signals that did not retain significance after Bonferroni correction), together with significant hematologic toxicity, further limiting its consideration as a viable option (16).

Based on our findings, Pola-R-CHP may be considered a potentially promising treatment option, pending prospective confirmation, for DEL patients. However, given that this evidence derives from subgroup analyses and lacks confirmatory OS benefit data, clinical decision-making should incorporate individual patient factors. The NCCN guidelines have included Pola-R-CHP as a category 1 recommendation for DLBCL, but specific guidance for the DEL subpopulation remains absent (30). CR-CHOP, from the only trial (DEB) designed specifically for DEL patients, showed nonsignificant PFS and OS trends that warrant further investigation, particularly in East Asian populations (14).

An important consideration for the transitivity assumption underlying this NMA is the geographic and ethnic heterogeneity among the included trials. The DEB trial enrolled exclusively Chinese patients, whereas POLARIX and CAVALLI enrolled global cohorts, and REMoDL-B enrolled predominantly European patients. Pharmacogenomic differences in drug metabolism, particularly CYP3A4/5 polymorphisms affecting vincristine and doxorubicin clearance, as well as differences in supportive care protocols and healthcare infrastructure, could serve as effect modifiers and introduce clinical heterogeneity. Several factors mitigate this concern: R-CHOP, the common comparator, yielded consistent DEL outcomes across trials (2-year PFS 40%–55%), suggesting broadly comparable disease behavior; the IHC-based DEL definition (MYC ≥ 40%, BCL2 ≥ 50%) represents a biologically coherent mechanism independent of ethnicity; and the DEB trial used standard R-CHOP dosing identical to international protocols. Nevertheless, the influence of ethnic and geographic factors on the relative treatment effect of CR-CHOP (tucidinostat) cannot be fully excluded, and caution is warranted when extrapolating the DEB results to non-Asian populations. This concern is particularly relevant for tucidinostat, an orally administered HDAC inhibitor that undergoes hepatic metabolism; recognized racial polymorphisms in drug-metabolizing enzymes may influence its systemic exposure and tolerability, further supporting caution when generalizing the CR-CHOP findings beyond the predominantly Chinese DEB population. Since the DEL phenotype, defined by MYC/BCL2 immunohistochemical coexpression, is biologically consistent across populations, these geographic and ethnic factors are expected to affect the extrapolation of the relative treatment effect rather than the internal validity of the within-network estimate. A cross-regional, DEL-specific prospective trial would help to confirm the generalizability of these findings.

This study has several important limitations. First, the network is star-shaped without closed loops, precluding consistency assessment between direct and indirect evidence; all inter-experimental comparisons are purely indirect, and a supplementary Bayesian NMA demonstrated that credible intervals under a random-effects model were wider than frequentist confidence intervals, underscoring the inherent uncertainty in these indirect comparisons. Nevertheless, NMA provides advantages over simple pairwise indirect comparisons by offering a unified effect-size estimation framework, enabling SUCRA-based ranking, and potentially improving precision through information borrowing across the network (17). Second, each comparison relies on a single study, rendering statistical estimation of between-study heterogeneity impossible. Third, the CAVALLI study’s use of historical controls does not fully satisfy the transitivity assumption underlying NMA, given potential differences in enrollment periods, supportive care standards, and DEL diagnostic workflows; however, a sensitivity analysis excluding CAVALLI confirmed that our primary conclusions are not contingent on this nonrandomized comparison (**Supplementary Table 2**). Fourth, safety data were derived from ITT populations rather than DEL subgroups; this extrapolation constitutes an ecological fallacy in the strict sense (31), as DEL patients typically present with higher IPI scores and more advanced disease, which could modify absolute toxicity rates compared with the broader DLBCL population. The safety NMA evaluated multiple AEs across five regimens; we therefore applied a Bonferroni correction (**Supplementary Table 3**). Only the Ven-R-CHOP signals (neutropenia, febrile neutropenia, anemia, and thrombocytopenia, together with any grade 3–4 adverse event and treatment discontinuation due to adverse events) and the ibrutinib hematologic signals (febrile neutropenia, anemia, and thrombocytopenia) retained significance after adjustment, whereas the remaining nominally significant findings—particularly those based on small samples or extreme OR values—are interpreted as exploratory and hypothesis-generating. Fifth, efficacy estimates rely on subgroup-level data, which are susceptible to ecological bias and may not reflect individualized treatment effects (31). Sixth, the DEB trial’s exclusively Chinese enrollment may limit the generalizability of CR-CHOP results to non-Asian populations. Seventh, the OS comparisons were characterized by wide confidence intervals for several regimens [for example, VR-CHOP, 0.83 (0.31–2.20); Ven-R-CHOP, 0.95 (0.50–1.80)], indicating limited statistical power; consequently, no firm conclusions regarding overall survival can be drawn, and these OS estimates should be regarded as exploratory.

In conclusion, Pola-R-CHP is currently the only regimen demonstrating a significant PFS benefit in DEL subgroup analyses, with the potential to influence clinical practice. However, given that this conclusion is based on subgroup analyses without confirmatory OS data, it cannot be directly recommended as the standard first-line regimen for all DEL patients. Prospective trials specifically designed for the DEL population, with OS as the primary endpoint, are urgently needed. Future studies should also investigate the role of molecular biomarkers such as TP53 mutations (32) and cell-of-origin classification in refining treatment selection for this high-risk population.

## Data Availability

The original contributions presented in the study are included in the article/**Supplementary Material**. The REMoDL-B individual-level data analyzed in this study are publicly available from the Gene Expression Omnibus under accession GSE117556. Further inquiries can be directed to the corresponding author.

## Supplementary material

Supplementary Table 1 Completed PRISMA-NMA reporting checklist.

Supplementary Table 2 Comparison of frequentist and Bayesian network meta-analysis results (fixed-effect and random-effects estimates), with model specifications.

Supplementary Table 3 Multiplicity-adjusted (Bonferroni) safety comparisons across the 47 grade 3–4 adverse-event comparisons.

Supplementary Figure 1 Comparison-adjusted funnel plots for progression-free survival and overall survival.
