# Supplementary figures and images for: Network meta-analysis of first-line R-CHOP-based regimens in MYC/BCL2 double-expressor diffuse large B-cell lymphoma
Source: Front Immunol. 2026 Jun 23;17:1832980. doi: 10.3389/fimmu.2026.1832980 (PMC13337834; doi:10.3389/fimmu.2026.1832980)

(A) PFS

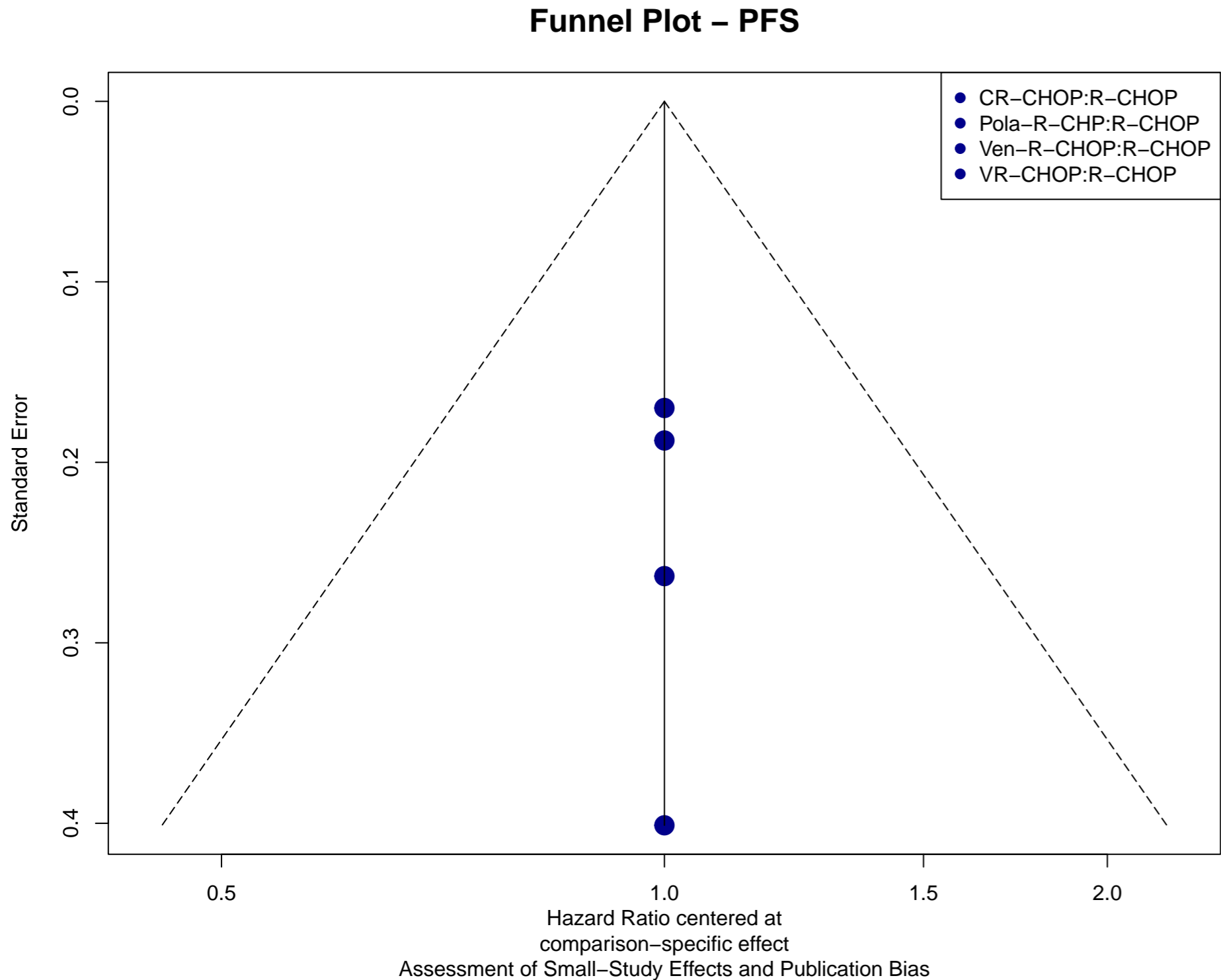

(B) OS

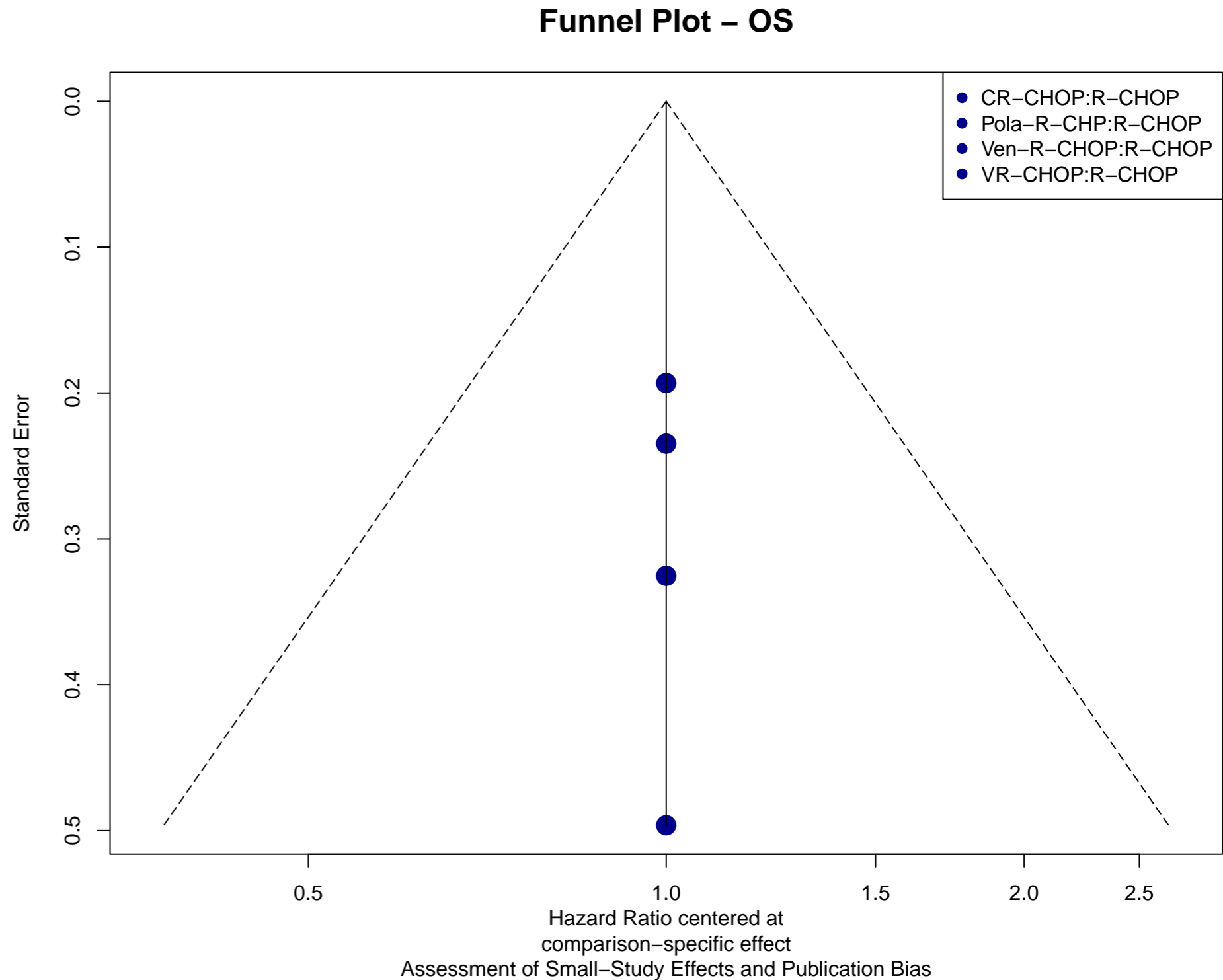

Supplement: Supplementary Table 1 — Completed PRISMA-NMA reporting checklist. [file DataSheet1.pdf]
