# Supplementary material for: Network meta-analysis of first-line R-CHOP-based regimens in MYC/BCL2 double-expressor diffuse large B-cell lymphoma
Source: Front Immunol. 2026 Jun 23;17:1832980. doi: 10.3389/fimmu.2026.1832980 (PMC13337834; doi:10.3389/fimmu.2026.1832980)
Supplement: Supplementary file 2 [file Table1.docx]

**Supplemental Table S1. PRISMA-NMA Checklist**

| **Section/Topic** | **#** | **Checklist Item** | **Reported on page/section** |
| --- | --- | --- | --- |
| Title | 1 | Identify the report as a systematic review incorporating a network meta-analysis (or related form of meta-analysis) | Title page |
| **ABSTRACT** |  |  |  |
| Structured summary | 2 | Provide a structured summary including, as applicable: background; objectives; data sources; study eligibility criteria, participants, and interventions; study appraisal and synthesis methods; results; limitations; conclusions and implications of key findings; systematic review registration number | Abstract |
| **INTRODUCTION** |  |  |  |
| Rationale | 3 | Describe the rationale for the review in the context of what is already known | Introduction, ¶1–4 |
| Objectives | 4 | Provide an explicit statement of questions being addressed, with reference to participants, interventions, comparisons, outcomes, and study design (PICOS) | Introduction, ¶5 |
| **METHODS** |  |  |  |
| Protocol and registration | 5 | Indicate whether a review protocol exists and if and where it can be accessed; and, if available, provide registration information, including registration number | Methods, Study design |
| Eligibility criteria | 6 | Specify study characteristics (eg, PICOS, length of follow-up) and report characteristics (eg, years considered, language, publication status) used as criteria for eligibility, giving rationale | Methods, Eligibility criteria |
| Information sources | 7 | Describe all information sources in the search and date last searched | Methods, Literature search |
| Search | 8 | Present full electronic search strategy for at least one database, including any limits used, such that it could be repeated | Supplemental Methods |
| Study selection | 9 | State the process for selecting studies | Methods, Eligibility criteria |
| Data collection process | 10 | Describe method of data extraction from reports and any processes for obtaining and confirming data from investigators | Methods, Data extraction |
| Data items | 11 | List and define all variables for which data were sought and any assumptions and simplifications made | Methods, Interventions and outcomes |
| Geometry of the network | S1 | Describe methods used to explore the geometry of the treatment network under study and potential biases related to it. This should include how the evidence base has been graphically summarized for presentation, and what characteristics were compiled and used to describe the evidence base to readers | Methods, Statistical analysis; Figure 2 |
| Risk of bias within individual studies | 12 | Describe methods used for assessing risk of bias of individual studies (including specification of whether this was done at the study or outcome level), and how this information is to be used in any data synthesis | Methods, Data extraction (RoB 2) |
| Summary measures | 13 | State the principal summary measures (eg, risk ratio, difference in means). Also describe the use of additional summary measures assessed, such as treatment rankings and surface under the cumulative ranking curve (SUCRA) values, as well as modified approaches used to present summary findings from meta-analyses | Methods, Statistical analysis |
| Planned methods of analysis | 14 | Describe the methods of handling data and combining results of studies for each network meta-analysis. This should include, but not be limited to, the following: handling of multiarm trials; selection of variance structure; selection of prior distributions in Bayesian analyses; and assessment of model fit | Methods, Statistical analysis (frequentist and Bayesian NMA) |
| Assessment of inconsistency | S2 | Describe the statistical methods used to evaluate the agreement of direct and indirect evidence in the treatment comparisons (consistency). Describe efforts taken to address its presence when found | Methods, Statistical analysis; Results, Bias assessment (star-shaped network precludes consistency testing) |
| Risk of bias across studies | 15 | Specify any assessment of risk of bias that may affect the cumulative evidence (eg, publication bias, selective reporting within studies) | Methods, Statistical analysis (funnel plots); Results, Bias assessment |
| Additional analyses | 16 | Describe methods of additional analyses if done, including sensitivity analyses. Describe which additional analyses were pre-specified | Methods, REMoDL-B DEL ascertainment; Methods, Statistical analysis (Bayesian NMA, CAVALLI exclusion); Results, Sensitivity analyses; Supplemental Table S2 |
| **RESULTS** |  |  |  |
| Study selection | 17 | Give numbers of studies screened, assessed for eligibility, and included in the review, with reasons for exclusions at each stage, ideally with a flow diagram | Results, Study selection; Figure 1 |
| Presentation of network structure | S3 | Provide a network graph of the included studies to enable visualization of the geometry of the treatment network | Figure 2 |
| Summary of network geometry | S4 | Provide a brief overview of characteristics of the treatment network. This may include commentary on the abundance of trials and randomized patients for the different interventions and pairwise comparisons in the network, gaps of evidence in the treatment network, and potential biases reflected by the network structure | Results, Study selection (¶2) |
| Study characteristics | 18 | For each study, present characteristics for which data were extracted and provide the citations | Table 1; Results, Study selection |
| Risk of bias within studies | 19 | Present data on risk of bias of each study and, if available, any outcome-level assessment | Methods, Data extraction (RoB 2); Table 4 (RoB column) |
| Results of individual studies | 20 | For all outcomes considered (benefits or harms), present for each study: simple summary data for each intervention group and effect estimates and confidence intervals. Modified approaches may be needed to deal with information from larger networks | Table 2; Table 3; Figures 3–5 |
| Synthesis of results | 21 | Present results of each meta-analysis done, including confidence/credible intervals. In larger networks, authors may present pairwise comparisons against a chosen intervention in a forest plot and leading tables showing all possible pairwise comparisons in a network. Relative rankings may also be presented | Results, PFS/OS/Safety; Figures 3–5; Table 2; Figure 4 |
| Exploration for inconsistency | S5 | Describe results from investigations of inconsistency. This may include such information as measures of model fit to compare consistency and inconsistency models, P values from statistical tests, or summary of inconsistency estimates from different parts of the treatment network | Results, Bias assessment (not applicable: star-shaped network, no closed loops) |
| Risk of bias across studies | 22 | Present results of any assessment of risk of bias across studies for the evidence base being studied | Results, Bias assessment; supplemental Figure S1 (funnel plots) |
| Results of additional analyses | 23 | Give results of additional analyses, if done | Results, Sensitivity analyses; Supplemental Table S2 |
| **DISCUSSION** |  |  |  |
| Summary of evidence | 24 | Summarize the main findings, including the strength of evidence for each main outcome; consider their relevance to key groups | Discussion, ¶1–2 |
| Limitations | 25 | Discuss limitations at study and outcome level (eg, risk of bias), and at review level (eg, incomplete retrieval of identified research, reporting bias) | Discussion, ¶6 (limitations) |
| Conclusions | 26 | Provide a general interpretation of the results in the context of other evidence, and implications for future research | Discussion, ¶7 |
| FUNDING | 27 | Describe sources of funding for the systematic review and other support; role of funders for the systematic review. This should also include information regarding whether funding has been received from manufacturers of treatments in the network and/or whether some of the authors are employed by manufacturers | Acknowledgments |

*S = items specific to network meta-analysis (PRISMA-NMA extension items).*
