# Supplementary material for: Network meta-analysis of first-line R-CHOP-based regimens in MYC/BCL2 double-expressor diffuse large B-cell lymphoma
Source: Front Immunol. 2026 Jun 23;17:1832980. doi: 10.3389/fimmu.2026.1832980 (PMC13337834; doi:10.3389/fimmu.2026.1832980)
Supplement: Supplementary file 4 [file Table3.docx]

**Supplementary Table S3. Multiplicity-adjusted (Bonferroni) safety comparisons.**

Family of comparisons N = 47 (all grade 3–4 adverse-event × regimen comparisons reported in Table 3). The Bonferroni-adjusted significance threshold is 0.05 / 47 = 0.00106. Nominal significance is defined as P < 0.05 (marked * in Table 3); comparisons retaining significance after Bonferroni correction (P < 0.00106) are marked † in Table 3.

| **Adverse event** | **Regimen** | **OR (95% CI)** | **P** | **Nominal (P<0.05)** | **After Bonferroni (P<0.00106)** |
| --- | --- | --- | --- | --- | --- |
| Neutropenia | CR-CHOP | 1.86 (1.27–2.74) | 0.0016 | Yes | No |
| Neutropenia | Ibrutinib+R-CHOP | 0.76 (0.58–0.99) | 0.0446 | Yes | No |
| Neutropenia | Pola-R-CHP | 0.88 (0.66–1.18) | 0.3858 | — | — |
| Neutropenia | VR-CHOP | 1.20 (0.92–1.56) | 0.1708 | — | — |
| Neutropenia | Ven-R-CHOP | 3.42 (2.43–4.80) | <0.001 | Yes | **Yes** |
| Febrile neutropenia | CR-CHOP | 1.35 (0.46–3.97) | 0.5800 | — | — |
| Febrile neutropenia | Ibrutinib+R-CHOP | 1.96 (1.39–2.78) | <0.001 | Yes | **Yes** |
| Febrile neutropenia | Pola-R-CHP | 1.84 (1.19–2.86) | 0.0070 | Yes | No |
| Febrile neutropenia | VR-CHOP | 0.95 (0.65–1.39) | 0.8017 | — | — |
| Febrile neutropenia | Ven-R-CHOP | 2.26 (1.56–3.28) | <0.001 | Yes | **Yes** |
| Anemia | CR-CHOP | 2.13 (1.21–3.75) | 0.0091 | Yes | No |
| Anemia | Ibrutinib+R-CHOP | 2.15 (1.45–3.19) | <0.001 | Yes | **Yes** |
| Anemia | Pola-R-CHP | 1.47 (0.94–2.29) | 0.0883 | — | — |
| Anemia | VR-CHOP | 0.73 (0.36–1.48) | 0.3875 | — | — |
| Anemia | Ven-R-CHOP | 3.29 (2.14–5.07) | <0.001 | Yes | **Yes** |
| Peripheral neuropathy | Ibrutinib+R-CHOP | 5.17 (1.49–18.01) | 0.0098 | Yes | No |
| Peripheral neuropathy | Pola-R-CHP | 1.42 (0.45–4.50) | 0.5602 | — | — |
| Peripheral neuropathy | VR-CHOP | 2.72 (0.72–10.30) | 0.1420 | — | — |
| Vomiting | Ibrutinib+R-CHOP | 3.60 (1.18–11.04) | 0.0248 | Yes | No |
| Vomiting | Pola-R-CHP | 1.69 (0.40–7.10) | 0.4803 | — | — |
| Vomiting | VR-CHOP | 1.87 (0.68–5.09) | 0.2226 | — | — |
| Vomiting | Ven-R-CHOP | 9.88 (2.04–47.98) | 0.0045 | Yes | No |
| Nausea | Ibrutinib+R-CHOP | 4.46 (1.26–15.78) | 0.0203 | Yes | No |
| Nausea | Pola-R-CHP | 2.53 (0.49–13.14) | 0.2702 | — | — |
| Nausea | VR-CHOP | 0.33 (0.09–1.23) | 0.0990 | — | — |
| Nausea | Ven-R-CHOP | 9.88 (2.04–47.98) | 0.0045 | Yes | No |
| Sepsis | Pola-R-CHP | 0.33 (0.03–3.22) | 0.3412 | — | — |
| Sepsis | VR-CHOP | 1.23 (0.60–2.53) | 0.5710 | — | — |
| Sepsis | Ven-R-CHOP | 2.31 (0.70–7.66) | 0.1699 | — | — |
| Any grade 3–4 AE | Pola-R-CHP | 1.04 (0.79–1.36) | 0.8578 | — | — |
| Any grade 3–4 AE | VR-CHOP | 1.41 (1.02–1.94) | 0.0385 | Yes | No |
| Any grade 3–4 AE | Ven-R-CHOP | 3.26 (2.11–5.03) | <0.001 | Yes | **Yes** |
| Treatment-related deaths | Ibrutinib+R-CHOP | 1.53 (0.73–3.22) | 0.2621 | — | — |
| Treatment-related deaths | Pola-R-CHP | 1.32 (0.57–3.04) | 0.5237 | — | — |
| Treatment-related deaths | VR-CHOP | 0.67 (0.19–2.38) | 0.5344 | — | — |
| Treatment-related deaths | Ven-R-CHOP | 0.44 (0.17–1.16) | 0.0965 | — | — |
| Thrombocytopenia | CR-CHOP | 2.26 (1.37–3.74) | 0.0015 | Yes | No |
| Thrombocytopenia | Ibrutinib+R-CHOP | 2.92 (1.75–4.86) | <0.001 | Yes | **Yes** |
| Thrombocytopenia | VR-CHOP | 2.05 (0.82–5.12) | 0.1258 | — | — |
| Thrombocytopenia | Ven-R-CHOP | 17.24 (8.25–36.01) | <0.001 | Yes | **Yes** |
| Lymphocyte count decreased | CR-CHOP | 1.15 (0.76–1.72) | 0.5100 | — | — |
| Lymphocyte count decreased | Ibrutinib+R-CHOP | 1.13 (0.70–1.81) | 0.6150 | — | — |
| Pneumonia | CR-CHOP | 2.43 (1.28–4.63) | 0.0066 | Yes | No |
| Pneumonia | Ibrutinib+R-CHOP | 2.57 (1.26–5.25) | 0.0097 | Yes | No |
| Pneumonia | Ven-R-CHOP | 0.87 (0.41–1.89) | 0.7325 | — | — |
| Discontinuation due to AE | Ibrutinib+R-CHOP | 1.00 (0.02–50.76) | 0.9981 | — | — |
| Discontinuation due to AE | Ven-R-CHOP | 2.91 (1.91–4.43) | <0.001 | Yes | **Yes** |

**Summary.** Of the comparisons nominally significant at P < 0.05, the following nine retain significance after Bonferroni correction: neutropenia (Ven-R-CHOP); febrile neutropenia (ibrutinib+R-CHOP and Ven-R-CHOP); anemia (ibrutinib+R-CHOP and Ven-R-CHOP); thrombocytopenia (ibrutinib+R-CHOP and Ven-R-CHOP); any grade 3–4 AE (Ven-R-CHOP); and discontinuation due to AE (Ven-R-CHOP). All remaining nominally significant comparisons do not retain significance after correction and are interpreted as exploratory and hypothesis-generating.

*Safety analyses were not the primary endpoint; the multiplicity adjustment is presented for transparency, while recognizing that for harm detection high sensitivity is also clinically important. POLARIX (Pola-R-CHP) odds ratios are computed on the corrected safety-evaluable population (435/438), consistent with Table 3.*
